# Neurons innervating both the central amygdala and the ventral tegmental area encode different emotional valences

**DOI:** 10.3389/fnins.2023.1178693

**Published:** 2023-05-05

**Authors:** Anqi Liu, Yuelin Cheng, Ju Huang

**Affiliations:** ^1^Center for Brain Science of Shanghai Children’s Medical Center, Shanghai Jiao Tong University School of Medicine, Shanghai, China; ^2^Department of Anatomy and Physiology, Shanghai Jiao Tong University School of Medicine, Shanghai, China; ^3^Jeffrey Trail Middle School, Irvine, CA, United States

**Keywords:** emotional valence, viral tracing, optogenetic activation, central amygdala (CeA), ventral tegmental area (VTA), posterior bed nucleus of the stria terminalis (pBNST), pedunculopontine tegmental nucleus (PPTg)

## Abstract

Mammals are frequently exposed to various environmental stimuli, and to determine whether to approach or avoid these stimuli, the brain must assign emotional valence to them. Therefore, it is crucial to investigate the neural circuitry mechanisms involved in the mammalian brain’s processing of emotional valence. Although the central amygdala (CeA) and the ventral tegmental area (VTA) individually encode different or even opposing emotional valences, it is unclear whether there are common upstream input neurons that innervate and control both these regions, and it is interesting to know what emotional valences of these common upstream neurons. In this study, we identify three major brain regions containing neurons that project to both the CeA and the VTA, including the posterior bed nucleus of the stria terminalis (pBNST), the pedunculopontine tegmental nucleus (PPTg), and the anterior part of the basomedial amygdala (BMA). We discover that these neural populations encode distinct emotional valences. Activating neurons in the pBNST produces positive valence, enabling mice to overcome their innate avoidance behavior. Conversely, activating neurons in the PPTg produces negative valence and induces anxiety-like behaviors in mice. Neuronal activity in the BMA, on the other hand, does not influence valence processing. Thus, our study has discovered three neural populations that project to both the CeA and the VTA and has revealed the distinct emotional valences these populations encode. These results provide new insights into the neurological mechanisms involved in emotional regulation.

## Introduction

Emotions are effectively defined as states elicited by rewards or punishments (Rolls, 2021). Emotional valence is the “sign” of the states of the central nervous system to respond to different environmental stimuli, facilitating the creatures to determine whether these stimuli are rewarding or aversive (Lammel et al., 2012). Positive valence represents rewarding states, which are typically displayed as approaching behavior. Negative valence represents aversive states, which are typically displayed as avoidance behavior (Tye, 2018). Therefore, the neural circuitry mechanisms involved in emotional valence processing in the mammalian brain are worthy of investigation.

The ventral tegmental area (VTA) in rodents primarily mediates reward, motivated behaviors, and the processing of salient sensory information (Fields et al., 2007; Grace et al., 2007; Bromberg-Martin et al., 2010; Meye and Adan, 2014; Nikulina et al., 2014; Overton et al., 2014). In contrast, the central amygdala (CeA) in rodents primarily regulates anxiety, fear-associated learning, pain control, and defensive behaviors evoked by threat stimuli (Hunt and Mantyh, 2001; Ehrlich et al., 2009; Ciocchi et al., 2010; Tye et al., 2011; Tovote et al., 2015). As the CeA and VTA are both critical brain regions involved in emotional regulation with nearly opposite functions, we were curious whether these regions share any common upstream inputs. Considering the existence that a population of calcitonin gene-related peptide (CGRP) neurons in the lateral parabrachial nucleus (LPB) innervating both the CeA and the VTA (Han et al., 2015; Qiao et al., 2019), we suspected that there might be other brain regions also containing neurons that innervated both the CeA and the VTA. Thus, it is interesting to investigate the existence of such common upstream inputs and the emotional valences they encode.

In the present study, we searched for the common upstream input neurons of the CeA and the VTA using the intersection-subtraction (IS) reporter mice, in which the neurons containing both Cre and Flp recombinases are labeled by green fluorescent protein (GFP; He et al., 2016). In addition to the LPB, we have identified three major brain regions that contain neurons innervating both the CeA and the VTA. We employed optogenetic stimulation and behavioral tests to confirm that these three neural populations encode different emotional valences. These findings provide new insights into the neurological mechanisms of emotional regulation.

## Results

### Neural populations that innervate both the CeA and the VTA

**Figure 1:**
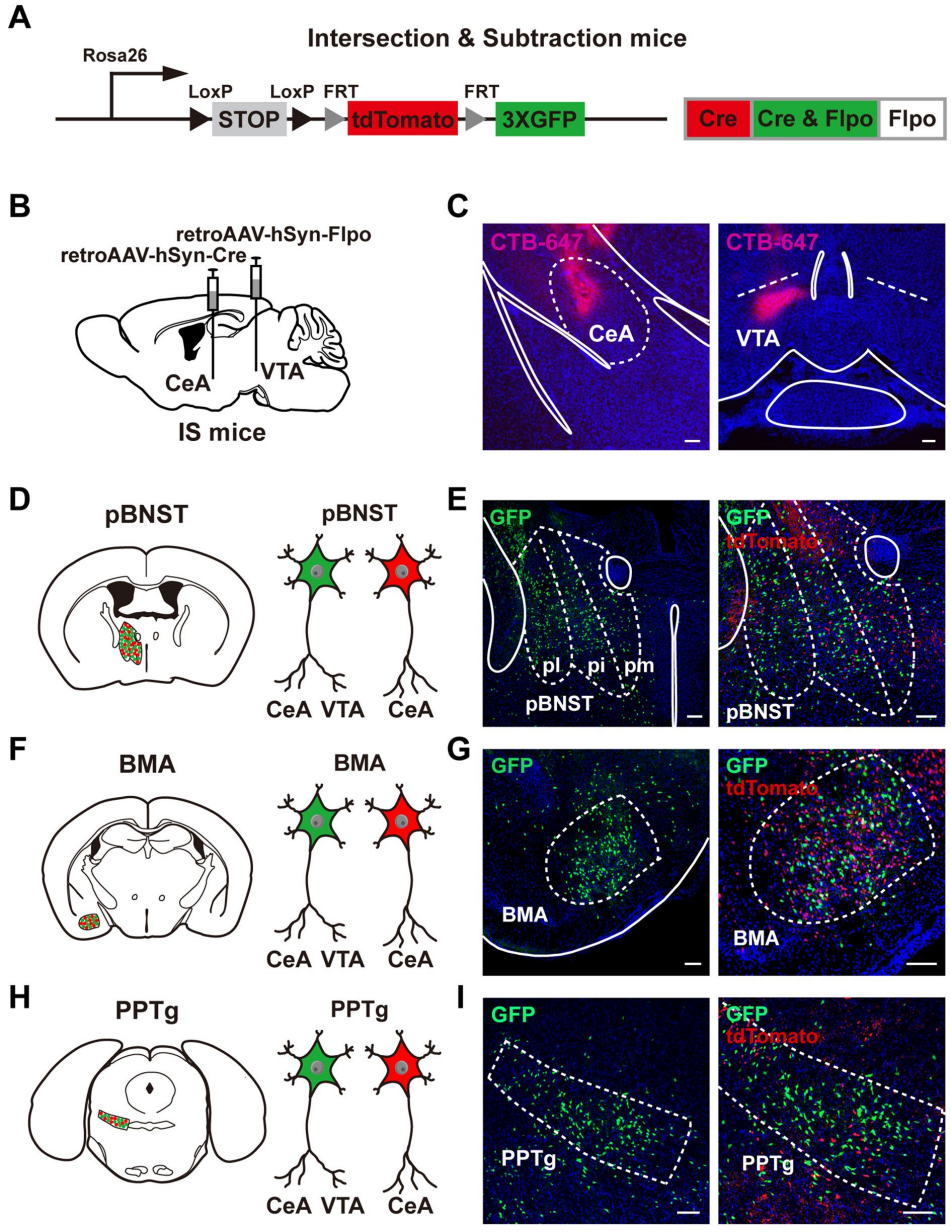
Neural populations that innervate both the CeA and the VTA. **(A)** A schematic diagram of the intersection-subtraction (IS) mice. **(B)** A schematic diagram of viral injection using AAV vector virus to retrogradely trace the neurons innervating the CeA and the VTA in IS mice. **(C)** The injection sites of the CeA and the VTA using virus mixed with CTB-647. Scale bars, 100 μm. **(D–I)** Three brain regions contained GFP-expressing neurons projecting to both the CeA and the VTA, including the pBNST **(D,E)**, the BMA **(F,G)**, and the PPTg **(H,I)**. **(E,G,I)** Left: position images of the GFP-positive neuronal populations. Right: images of the tdTomato-positive neurons alongside the GFP-positive neurons. Scale bars, 100 μm.

To search for neurons that innervate both the CeA and the VTA, we used the intersection-subtraction (IS) reporter mice combined with viral tracing. In IS reporter mice, to enable the GFP-positive neurons to represent neurons that project to both the CeA and the VTA, and the tdTomato-positive neurons to represent neurons that project to the CeA only (He et al., 2016; Figure 1A), we delivered the retrogradely transported adeno-associated virus (retroAAV-hSyn-Cre) that expressed Cre recombinase into the CeA, and the retroAAV-hSyn-Flpo that expressed Flpo recombinase into the VTA of IS reporter mice, respectively (Figure 1B). To indicate the accuracy of injection sites, we mixed the cholera toxin beta-subunit conjugated to a 647 fluorophore (CTB-647) with the viruses. We injected the retroAAV-hSyn-Cre with the CTB-647 into the CeA and the retroAAV-hSyn-Flpo with the CTB-647 into the VTA (Figures 1B,C). The mice with inaccurate viral injections were rejected.

To demonstrate the reliability of our methodology, we showed the GFP-positive neurons and tdTomato-positive neurons in the LPB, a brain region containing common upstream input neurons to both the CeA and the VTA (Supplementary Figures S2G,H; Han et al., 2015; Qiao et al., 2019). Compared to the known brain regions that contain neurons projecting to the CeA, e.g., the medial prefrontal cortex (mPFC), the ventromedial hypothalamic nucleus (VMH), the dorsal raphe (DR), the paraventricular nucleus of the thalamus (PVT), the anterolateral part of BNST (alBNST), and the insular cortex (IC; Gungor and Pare, 2016; Ren et al., 2018; Lo et al., 2019; Keyes et al., 2020; Chen et al., 2021; Ma et al., 2021; Wang et al., 2022), we observed a similar pattern of the CeA-projecting tdTomato-positive neurons in the mPFC, the IC, the alBNST, the PVT, the VMH, and the DR in our study (Supplementary Figures S2A–F). These results validate our methodological reliability for using the IS reporter mice.

We performed frozen sectioning 3 weeks after viral injection. Notably, we identified three major brain regions containing neurons that innervated both the CeA and the VTA, including the posterior bed nucleus of the stria terminalis (pBNST; Figures 1D,E), the anterior part of the basomedial amygdala (BMA; Figures 1F,G), and the pedunculopontine tegmental nucleus (PPTg; Figures 1H,I). The pBNST includes three subregions, the posteromedial part of the bed nucleus of stria terminalis (pmBNST), the posterointermediate part of the bed nucleus of stria terminalis (piBNST), and the posterolateral part of the bed nucleus of stria terminalis (plBNST; Figure 1E). We observed the GFP-positive neurons in all these three subregions of the pBNST. TdTomato-positive neurons were also displayed alongside the GFP-positive neurons. The GFP-positive axon terminals were shown in the CeA and the VTA (Supplementary Figures S1A,B). Taken together, we reveal that the pBNST, the BMA, and the PPTg are three major brain regions that contain neurons innervating both the CeA and the VTA.

### Activation of the upstream input neurons innervating both the CeA and the VTA produces different emotional valences

**Figure 2:**
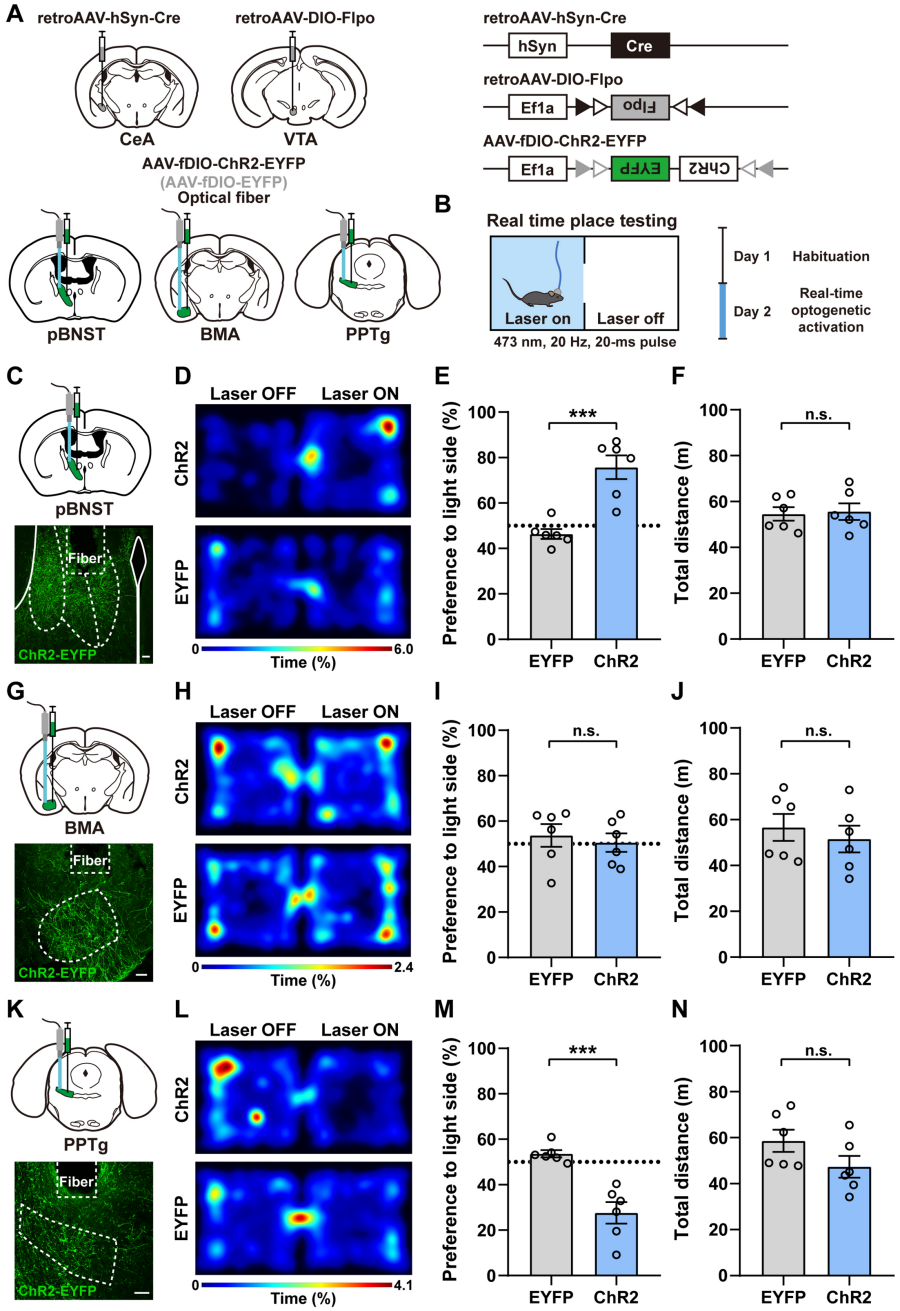
Activation of the upstream input neurons innervating both the CeA and the VTA produces different emotional valences. **(A)** A schematic diagram showing the viral injection and fiber implantation approach for optogenetic activation of neurons projecting to both the CeA and the VTA in wild-type mice. **(B)** The experimental protocol of the RTPT assay. **(C,G,K)** Expression of ChR2-EYFP and placement of the optic fiber (**C**, pBNST; **G**, BMA; **K**, PPTg). Scale bars, 100 μm. **(D,H,L)** Representative heatmaps of locomotor activity on the RTPT test day for the mice expressing ChR2 and EYFP (**D**, pBNST; **H**, BMA; **L**, PPTg). **(E,I,M)** Quantification of preference for the light side in ChR2 and EYFP mice (**E**, pBNST, ^***^p < 0.001; **I**, BMA; **M**, PPTg, ^***^p < 0.001; *n* = 6 mice for each group). **(F,J,N)** Quantification of locomotor activity in ChR2 and EYFP mice (**F**, pBNST; **J**, BMA; **N**, PPTg; *n* = 6 mice for each group). Data are presented as mean ± SEM.

**Figure 3:**
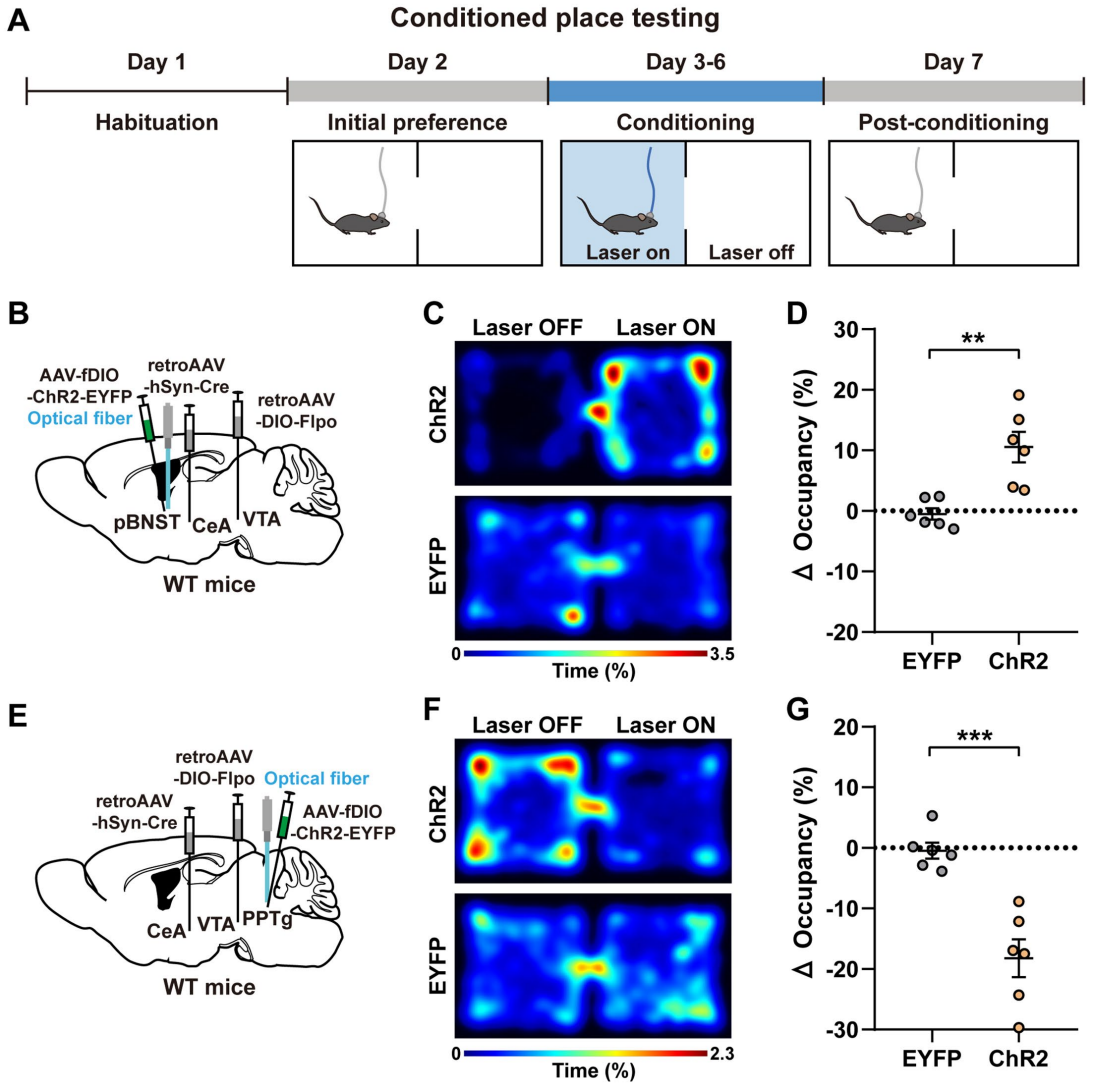
Activation of the pBNST and PPTg neurons are sufficient to evoke conditioned place preference and conditioned place aversion, respectively. **(A)** Experimental procedure of the CPT assay. **(B,E)** A schematic diagram of the viral injection and fiber implantation sites. **(C,F)** Representative heatmaps of locomotor activity on the sixth day of the experiment (i.e., the fourth day of conditioning) for the mice expressing ChR2 and EYFP (**C**, pBNST; **F**, PPTg). **(D,G)** Percent changes in the occupancy time on the stimulation side after conditioning (after conditioning—before conditioning; **D**, pBNST, ^**^p < 0.01; **G**, PPTg, ^***^p < 0.001; *n* = 6 mice for each group). Data are presented as mean ± SEM.

To examine whether activation of these neural populations produces different emotional valences, we used the real time place testing (RTPT) and conditioned place testing (CPT) paradigms to test whether these neurons encoded positive preference or aversive avoidance (Figures 2B, 3A; Zhao et al., 2019). We injected retroAAV-hSyn-Cre into the CeA and retroAAV-DIO-Flpo into the VTA in wild-type mice. We targeted the channelrhodopsin-2 (ChR2) to the upstream input neurons by delivering the AAV expressing Flpo-dependent ChR2 (AAV-fDIO-ChR2-EYFP, or AAV-fDIO-EYFP as the control) into the pBNST, the BMA, and the PPTg, respectively (Figure 2A). Neurons were activated by blue-laser light stimulation (473 nm, 4 mW, 20 Hz, and 20-ms pulse duration) through the implanted optic fiber above these brain regions. The behavioral experiments were performed 3 weeks after viral injection.

In the RTPT test, mice were placed in an apparatus with two identical chambers and allowed to freely explore the chamber for 20 min. One of the chambers was selected to be paired with a blue laser light. The optogenetic stimulation (473 nm, 4 mW, 20 Hz, and 20-ms pulse duration) was given when the mice entered the light-paired chamber and would stop immediately when the mice left the chamber. Compared with the control mice, the mice with ChR2-expressing neurons in the pBNST developed a consistent positive preference for the light-paired chamber, indicating that activation of the pBNST neurons was rewarding and associated with a positive valence (Figures 2C–E). In contrast, the mice with ChR2-expressing neurons in the PPTg displayed a strong negative avoidance for the light-paired chamber, indicating that activation of the PPTg neurons was aversive and produced a negative valence (Figures 2K–M). In addition, activating the ChR2-expressing neurons in the BMA had little or no effect on the preference or avoidance for the light-paired chamber (Figures 2G–I). As the control analysis, we found that activation of these upstream input neurons did not change the locomotor activities of the mice (Figures 2F,J,N). These findings suggest that activation of these upstream input neurons innervating both the CeA and the VTA produces distinct emotional valences.

Subsequently, we employed the conditioned place testing (CPT) paradigm to test whether optogenetic activation of these upstream neurons were sufficient to evoke conditioned place preference or conditioned place aversion (Figures 3A,B,E). Briefly, the CPT paradigm lasted 7 days. On the first day, each mouse was acclimated to the apparatus for 30 min. On the second day, the mice were allowed to freely explore the apparatus for 30 min, and the basal contextual preference of each mouse was measured. The conditioning period consisted of 4 days from the third to sixth day, with a 30-min session/per day. The mice were given the blue light stimulation (473 nm, 4 mW, 20 Hz, and 20-ms pulse duration) as soon as they entered the light-paired chamber, which was immediately stopped when they left. On the sixth day of the experiment (i.e., the fourth day of conditioning), the mice expressing ChR2 in the pBNST neurons displayed a positive preference to the light-paired chamber (Figure 3C). The mice expressing ChR2 in the PPTg neurons displayed a negative avoidance to the light-paired chamber (Figure 3F). On the last day (seventh day), no photostimulation was given. The mice were allowed to freely move and select chambers for 30 min. The post-conditioning preference was calculated as the change in the percentage of occupancy time in the light-stimulated chamber (after conditioning—before conditioning). We found that the mice expressing ChR2 in the pBNST neurons developed a profound preference for the chamber previously paired with the photostimulation, indicating an obvious positive valence encoded by these neurons (Figure 3D). In contrast, the mice expressing ChR2 in the PPTg neurons developed a marked avoidance for the chamber previously paired with the photostimulation, indicating a significant negative valence encoded by these neurons (Figure 3G). Activation of the BMA neurons did not change the basal contextual preference of mice (Supplementary Figures S3A,B). These results further demonstrate that these upstream input neurons innervating both the CeA and the VTA encode distinct emotional valences.

### Activation of the PPTg neurons innervating the CeA and the VTA induces anxiety-like behavior

**Figure 4:**
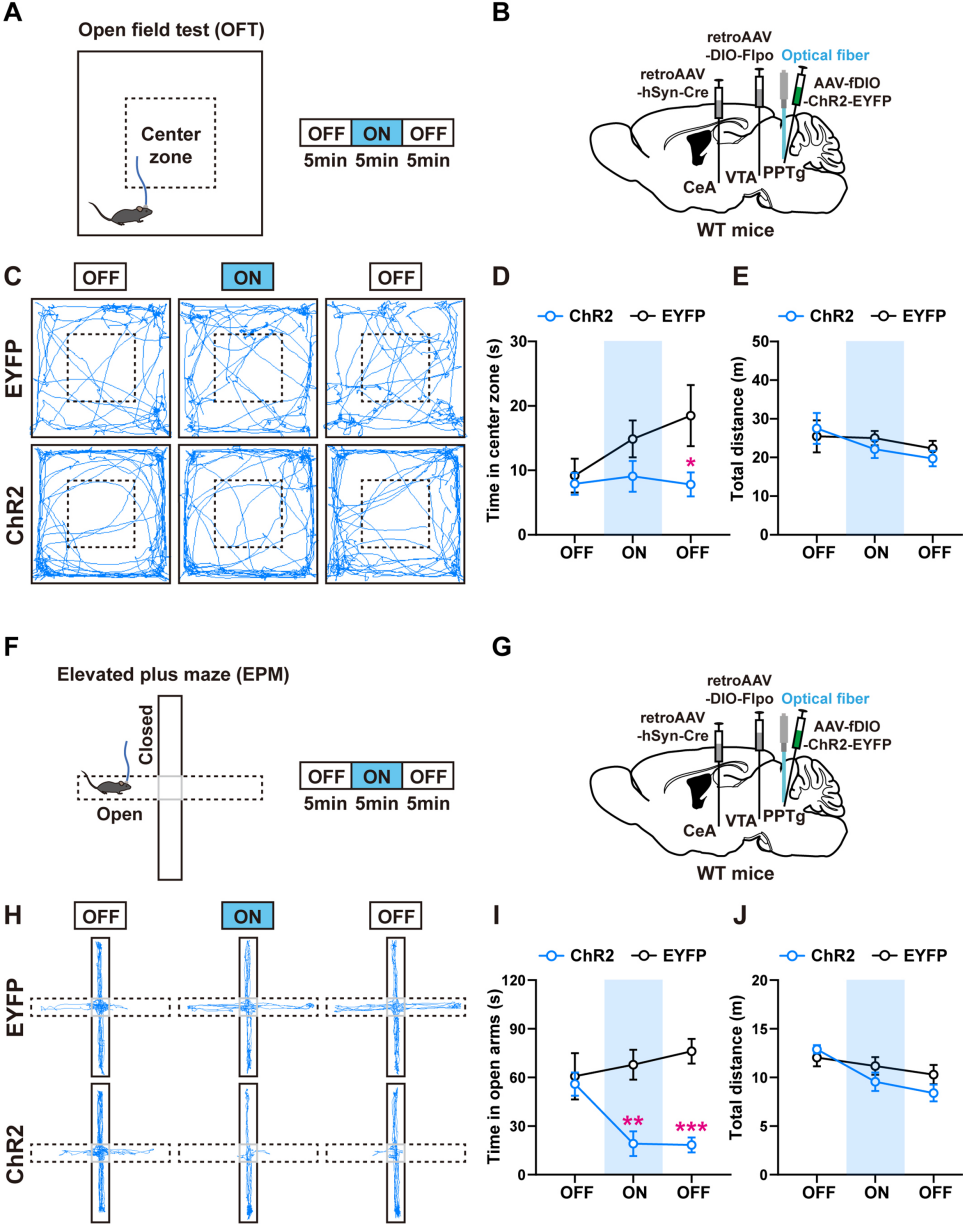
Activation of the PPTg neurons innervating the CeA and the VTA induces anxiety-like behavior. **(A)** Experimental procedure for intermittent optogenetic activation of ChR2-expressing neurons in the open field test. **(B)** A schematic diagram of the viral injection and fiber implantation sites. **(C)** Representative locomotor trajectories of mice expressing EYFP or ChR2 in the open field test. **(D,E)** Optogenetic activation of ChR2-expressing neurons in the PPTg reduced the time mice spent in the center zone but did not affect locomotor activity of mice in the open field test (EYFP: *n* = 6, ChR2: *n* = 7, time in center zone: ^*^p < 0.05 in 10–15 min OFF). **(F)** Experimental procedure for intermittent optogenetic activation of ChR2-expressing neurons in the elevated plus maze test. **(G)** A schematic diagram of the viral injection and fiber implantation sites. **(H)** Representative locomotor trajectories of mice expressing EYFP or ChR2 in the elevated plus maze test. **(I,J)** Optogenetic activation of ChR2-expressing neurons in the PPTg reduced the time mice spent in the open arms but did not affect locomotor activity of mice in the elevated plus maze test (EYFP: *n* = 6, ChR2: *n* = 6, time in open arms: ^**^p < 0.01 in 5–10 min ON, ^***^p < 0.001 in 10–15 min OFF). Data are presented as mean ± SEM.

To investigate whether the negative valence produced by the activation of PPTg neurons directly induces anxiety-like behaviors, we performed the anxiety-related behavioral analyses including the elevated plus maze (EPM) and the open field test (OFT; Figures 4A,B,F,G). Each mouse underwent a 15-min procedure in the anxiety-like behavioral tests (Figures 4A,F). The procedure contained three periods, and each period lasted 5 min. The mice were placed in the apparatus for free exploration in the first period to assess their baseline levels. Blue light stimulation (473 nm, 4 mW, 20 Hz, and 20-ms pulse duration) was applied to the mice in the second period. The mice continued to explore freely without light stimulation in the third period.

In the OFT, activation of the PPTg neurons had a tendency to reduce the time that the mice spent in the center zone, without changing their locomotor activity (Figures 4C–E). In the EPM, activation of the PPTg neurons decreased the time that the mice stayed in the open arms, without changing the total distance that the mice traveled in the whole apparatus (Figures 4H–J). These results indicate that activation of the PPTg neurons induces anxiety-related behaviors. In comparison, we found that activation of the BMA neurons had little or no effect on the anxiety-related behaviors (Supplementary Figures S3C–H).

### Activation of the pBNST neurons innervating the CeA and the VTA overcomes the innate avoidance behavior

**Figure 5:**
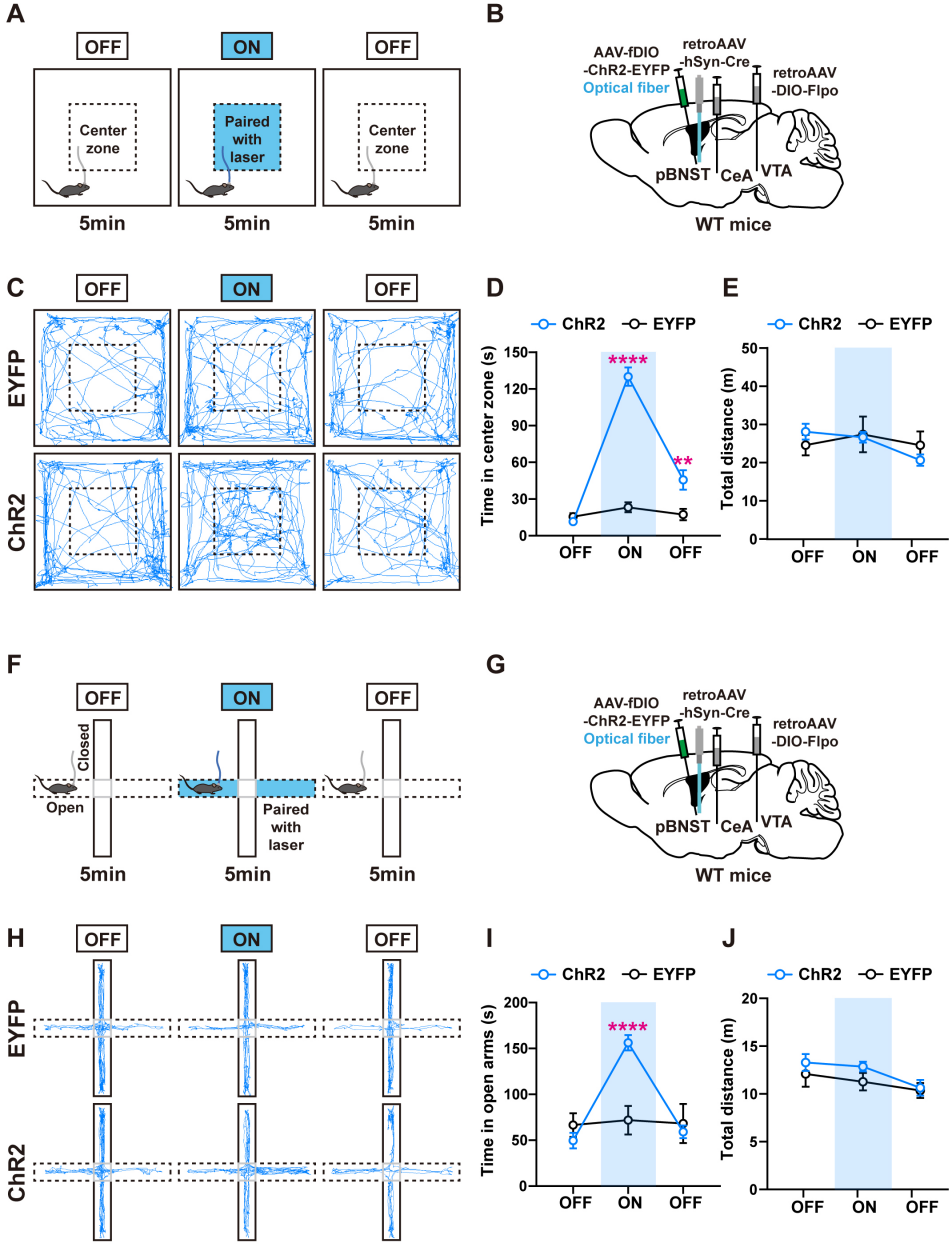
Activation of the pBNST neurons innervating the CeA and the VTA overcomes the innate avoidance behavior. **(A)** Experimental procedure for intermittent optogenetic activation of ChR2-expressing neurons in the modified open field test. Optogenetic activation is paired with the center zone and triggered when mice entered the center zone during 5–10 min. **(B)** A schematic diagram of the viral injection and fiber implantation sites. **(C)** Representative locomotor trajectories of mice expressing EYFP or ChR2 in the open field test. **(D,E)** ChR2-expressing mice spent more time in the stimulation-paired center zone and were more willing to explore the center zone after optogenetic stimulation than control mice, but locomotor activity was not affected (EYFP: *n* = 7, ChR2: *n* = 8, time in center zone: ^****^p < 0.0001 in 5–10 min ON, ^**^p < 0.01 in 10–15 min OFF). **(F)** Experimental procedure for intermittent optogenetic activation of ChR2-expressing neurons in the modified elevated plus maze. Optogenetic activation is paired with the open arms and triggered when mice enter these arms during 5–10 min. **(G)** A schematic diagram of the viral injection and fiber implantation sites. **(H)** Representative locomotor trajectories of mice expressing EYFP or ChR2 in the elevated plus maze test. **(I,J)** During 5–10 min, ChR2-expressing mice spent more time in the stimulation-paired open arms than control mice, but locomotor activity was not affected (EYFP: *n* = 6, ChR2: *n* = 8, time in open arms: ^****^p < 0.0001 in 5–10 min ON). Data are presented as mean ± SEM.

To investigate whether the positive valence produced by activating the pBNST neurons can help the mice overcome the innate avoidance behaviors, we performed the modified OFT and EPM tests (Hu et al., 2021; Figures 5A,B,F,G). The mice typically have an innate tendency to avoid places that give them negative valence. They prefer to stay in the outer perimeter of the open field rather than in the center zone. Thus, we paired light stimulation with the center zone that mice tended to avoid for 5 min after a baseline test (Figure 5A). A 473-nm blue light (4 mW, 20 Hz, and 20-ms pulse duration) was given immediately when the mouse entered the center zone, and the light stimulation was stopped immediately when the mouse left this center zone. In the elevated plus maze, the mice prefer to stay in the closed arms rather than explore the open arms. Thus, during the intermediate 5-min light-stimulation period, we paired the light stimulation with the open arms that the mice tended to avoid (Figure 5F).

In the modified OFT, we observed that the ChR2-expressing mice spent significantly more time in the center zone associated with light stimulation. We also found the mice were more willing to explore the center zone after optogenetic stimulation than the control mice (Figures 5C,D), indicating the positive conditioning effect. In the modified EPM test, the ChR2-expressing mice spent significantly more time in the open arms when we paired light stimulation with open arms (Figures 5H,I). There were no differences in the locomotor activity between the ChR2-expressing mice and the control mice (Figures 5E,J). These results suggest that activation of the pBNST neurons innervating the CeA and the VTA produces a strong positive valence, which is sufficient to help the mice overcome their innate avoidance behaviors.

## Discussion

The neural circuitry that mediates emotional valence processing in the central nervous system plays a crucial role for the survival of animals. Extensive research has been executed on the anatomical connectivity and emotional regulation by the CeA and the VTA individually. For example, the anterolateral part of BNST (alBNST) was known to project to the CeA (Gungor and Pare, 2016) and the other parts of the BNST project to the VTA (Jennings et al., 2013). The PPTg was known to project to the VTA (Yau et al., 2016) and the BMA project to the CeA (Petrovich et al., 1996). However, it is still not fully understood what neurons are the common upstream inputs that project to both the CeA and the VTA and what emotional valence these neurons encode. Combined virus-mediated retrograde tracing in the IS reporter mice and *in vivo* optogenetic activation, our study identified three major brain regions, the pBNST, the BMA, and the PPTg, containing neurons that innervated both the CeA and the VTA. The CeA/VTA-projecting neurons in the pBNST encode positive valence, and activation of them facilitates the mice to overcome their innate avoidance behaviors. In contrast, the CeA/VTA-projecting neurons in the PPTg encode negative valence, and activation of them caused anxiety-related behaviors in mice.

The PPTg is reported to regulate cognitive processing and locomotor activity (Steckler et al., 1994; Goetz et al., 2016; Syed et al., 2016; Takakusaki et al., 2016; Caggiano et al., 2018). Whether the PPTg brain region is involved in the regulation of anxiety emotions is controversial. Previous research on rats revealed that bilateral N-methyl-D-aspartate (NMDA)-induced lesions of the pontine tegmentum, which included the PPTg, increased anxiety (Leri and Franklin, 1998). Another study showed that bilateral electrolytic lesions of the PPTg produced slightly reduced anxiety-like behavior, which indicated a slight anxiolytic effect (Homs-Ormo et al., 2003). However, the large area of lesions caused by drugs or electrolytes were less precise in the anatomical localization of emotional state production. Anxiety-associated effects might be produced by lesions to regions adjacent to the PPTg. Here, our study used viral tracing and optogenetic activation and revealed that activation of the PPTg neurons projecting to the CeA and the VTA triggered negative emotions and increased anxiety-like behavior. These findings suggest that the PPTg is involved in the regulation of anxiety.

The BNST is the core of the brain’s emotion-processing network because it is a critical and distinctive processing junction with dense projection connections (Avery et al., 2014; Myers et al., 2014; Lebow and Chen, 2016). The BNST plays an essential role in anxiety-state regulation. Although recent research has shown anxiogenic and anxiolytic effects following the activation of different subregions in the anterior BNST (Jennings et al., 2013; Kim et al., 2013), little is known about how the posterior BNST (pBNST) contributes to emotional processing. A recent study showed that optogenetic stimulation of corticotropin-releasing factor receptor type 2 (CRFR2) neurons in the pBNST was involved in the modulation of behavioral responses to stressful situations (Henckens et al., 2017). The activation of these neurons immediately reduced the basal anxiety of mice, which was associated with an anxiolytic phenotype. In this study, we showed that optogenetic activation of the pBNST neurons projecting to the CeA and the VTA produced positive emotional valence that was sufficient to overcome the innate avoidance behaviors, suggesting that this rewarding effect is strong enough to resist the negative emotions. Thus, the pBNST seems to play a critical role in reversing negative emotions.

The BMA is a brain region that has yet to be studied. A previous study showed that different neural populations in the BMA were selectively activated in either the aversive or the safe environments (Adhikari et al., 2015), suggesting that the BMA might be involved in the regulation of emotional states. Our study found that the neurons in the BMA innervating both the CeA and VTA showed a neutral valence, neither the positive nor the negative valence. However, we cannot exclude the possibility that other neural populations in the BMA might be involved in emotional regulation.

In the open field test, we observed a trend for mice with light-activated PPTg neurons to spend less time in the center zone. However, during the light-on period, we failed to find the significant difference when compared with the control mice. During the light-off (10–15 min) period, we observed a significant decrease in the time spent in the center zone of the open field test and in the open arms of the elevated plus maze test for the mice with light-activated PPTg neurons (Figures 4D,I), as well as a significant increase in the time spent in the center zone for the mice with light-activated pBNST neurons (Figure 5D). We suspected that the observed effects during the light-off period might be caused by the continuing impact of neural activation in the light-on period. This might suggest that the mice were quickly conditioned by the light stimulation, either positively or negatively. Nevertheless, we could not exclude the possibility that the continuous effects during the light-off period stemmed from the indirect effect from the other neuronal network.

There were some limitations in our study. For instance, the traced neurons in our work were not precisely marked with molecular markers. By performing the smFISH in the IS reporter mice, we preliminarily explored their neuronal identity (excitatory vs. inhibitory) of these neurons. We found that 24.1 ± 1.8 and 67.7 ± 1.7% of GFP-positive neurons in the pBNST expressed the excitatory neuronal marker, *Vglut2*, and the inhibitory neuronal marker, *Vgat,* respectively (Supplementary Figure S4A). Meanwhile, 56.1 ± 1.5 and 34.9 ± 3.0% of GFP-positive neurons in the BMA expressed *Vglut2* and *Vgat,* respectively (Supplementary Figure S4B). In the PPTg, 92.5 ± 1.2 and 5.5 ± 0.5% of GFP-positive neurons expressed *Vglut2* and *Vgat,* respectively (Supplementary Figure S4C). However, further study is needed in the future to determine whether it is the excitatory or inhibitory neurons that mediate the positive or the negative valence. In addition, future research should focus on the pathways how these neurons modulate valence processing.

Taken together, our research identifies three neural populations that project to the CeA and the VTA, and these neurons encode distinct emotional valences. The pBNST and the PPTg may be important nodes in the neural circuitry network of emotional regulation. We believe that neurons in these brain regions provide potential targets for the treatment of mood disorders.

## Materials and methods

### Animals

The Institutional Animal Care and Use Committee at Shanghai Jiao Tong University School of Medicine approved all experimental protocols (Protocol A-2019-060). Mice were housed at a controlled temperature (25°C) with sufficient food and water and a 12-h day/12-h night cycle (daytime, 7 a.m. to 7 p.m.). The following mouse lines were used in this study: wild-type C57BL/6 J (SLAC Laboratory Animal, Shanghai) and Intersection-Subtraction (IS) reporter mice (Miao He’s laboratory at Fudan University). The experimental groups were randomly assigned to age-balanced littermate mice. Adult male mice aged 10–12 weeks were used for the behavioral tests. Our anatomical research used adult mice aged 8–10 weeks.

### Stereotaxic viral injection

Stereotaxic surgeries were performed under anesthesia (100 mg/kg sodium pentobarbital, intraperitoneally). The virus was injected in a volume of 250 nL/site at a flow rate of 25 nL/min using a stereotaxic injector controller. 15 min after the virus injection, the glass pipette was slowly removed to prevent virus reflux. For optogenetic stimulation experiments, an optical fiber (200 μm core, NA = 0.37) was implanted at the indicated sites. The fiber was attached to the skull with dental cement. We verified the accuracy of virus infection and optic fiber implantation after the experiments. The mice were allowed 3 weeks to recover from surgery and virus expression before the behavioral tests.

The corresponding coordinates of the target sites were used (calculated from bregma): VTA (AP, − 3.08 mm; ML, +0.50 mm; DV, −4.50 mm), CeA (AP, −1.46 mm; ML, +2.60 mm; DV, −4.60 mm), pBNST (AP, −0.22 mm; ML, +0.70 mm; DV, −4.50 mm), BMA (AP, −1.34 mm; ML, +2.40 mm; DV, −5.40 mm), and PPTg (AP, −4.55 mm; ML, +1.10 mm; DV, −3.75 mm). The following virus and titer were used: RetroAAV2/2-hSyn-Cre-WPRE-pA (Shanghai Taitool, 2.55E + 13 v.g./mL); Retro AAV2/2-hSyn-Flpo-WPRE-pA (Shanghai Taitool, 2.32E + 12 v.g./mL); RetroAAV2/2-Ef1a-DIO-Flpo-WPRE-hGH-pA (Brain VTA, 5.93E + 12 v.g./mL); AAV2/9-Ef1a-fDIO-hChR2-EYFP-WPRE-pA (Brain VTA, 5.26E + 12 v.g./mL); and AAV2/9-Ef1a-fDIO-EYFP-WPRE-pA (Brain VTA, 5.28E + 12 v.g./mL).

To rule out the possibility of leaky contamination, we mixed the CTB-647 with the viruses and performed *post hoc* verification by sectioning the tissues after the behavioral tests. The mice with incorrect or leaky injections were excluded from the data quantification. CTB-647 was purchased from Thermo Fisher Scientific and diluted to 10 μg/μL with sterile water.

### Optogenetic stimulation

We injected retro-AAV-hSyn-Cre into the CeA, retroAAV-DIO-Flpo into the VTA, and AAV-fDIO-ChR2-EYFP or AAV-fDIO-EYFP into the respective upstream target regions of wild-type mice. The optic fiber was implanted above the target regions to activate the upstream input neurons. The cell bodies of neurons were activated by 473-nm blue laser light (4 mW, 20 Hz, and 20-ms pulse duration). The cohorts of mice do all the following behavioral test consecutively (RTPT-CPT-OFT-EPM). After the experiment, we verified the accuracy of virus expression and fiber placement.

### Real time place testing

Mice in the real time place testing were placed in two identical chambers (each 25 cm × 25 cm × 25 cm) with a 5-cm wide opening in the middle. Mice were allowed to move freely throughout the apparatus for 20 min. When the mouse entered a chamber paired with the photostimulation, a constant light stimulation (473-nm blue laser light, 4 mW, 20 Hz, 20-ms pulse duration) was triggered. When the mouse entered the other chamber, the light stimulation ended. A video-tracking system (EthoVision 3.0, Noldus) was used to measure the time the mouse spent in each chamber and the total distance traveled during the 20-min trial.

### Conditioned place testing

Mice in the conditioned place testing were placed in two distinct chambers (each 25 cm × 25 cm × 25 cm) with different visual stripes (black vs. white) and textural cues (smooth transparent vs. white matte floor). The mice were acclimated to the apparatus for 30 min on the first day. The next day, mice were given free access to the two-chamber apparatus for 30 min to assess basal preference. The conditioning phase lasted 4 days (i.e., days 3–6), with 30 min of conditioning per day. Mice were given blue light stimulation (473 nm, 4 mW, 20 Hz, 20-ms pulse duration) when they entered the stimulation-paired chamber, and the stimulation was stopped immediately when the mice left the chamber. On day 7, the mice were allowed to move freely throughout the two-chamber apparatus for 30 min without photostimulation. The positions of the mice were tracked. A video-tracking system (EthoVision 3.0, Noldus) was used to monitor the position of the mice. The percent change in time occupied on the light-stimulated side (after conditioning—before conditioning) was analyzed.

### Open field test

Mice in the open field test (OFT) were placed in an open field area (40 cm × 40 cm × 30 cm) and allowed to explore for 15 min. The test consisted of three 5-min sessions (laser off–on–off). Blue light (473 nm, 20 Hz, and 20-ms pulse) was delivered to the target brain region during the stimulation period. Photostimulation was performed when mice entered the center zone in the modified open field test, and it was terminated immediately when the mice exited the center zone. A video-tracking system (EthoVision 3.0, Noldus) was used to measure the time spent in the center zone (20 cm × 20 cm) and the total distance in the entire device.

### Elevated plus maze

Mice in the elevated plus maze (EPM) test were placed in a device that consisted of a central square (7 cm × 7 cm), two open arms (30 cm × 7 cm), and two closed arms with walls (30 cm × 7 cm × 15 cm). Mice were placed in the central square facing an open arm and allowed to explore for 15 min. The test consisted of three 5-min sessions (a pre-stimulation period, a stimulation period, and a post-stimulation period). Mice were stimulated with 473-nm photostimulation (4 mW, 20 Hz, and 20-ms pulse) during the stimulation period. Photostimulation was performed when mice entered the open arms in the modified elevated plus maze test, and it was terminated immediately when the mice exited the open arms. A video tracking system (EthoVision 3.0, Noldus) was used to record the time spent in the open arms and the total distance traveled in the entire device.

### Histology and immunostaining

Mice were perfused with saline followed by 4% paraformaldehyde (PFA). Brains were post-fixed at 4°C for 2 h and cryopreserved overnight in phosphate buffered saline (PBS) with 30% sucrose until the tissue sank. The tissue was cut into 50 μm sections using a freezing microtome. Tissue sections were blocked in 3% BSA, 0.4% Triton X-100, and 5% goat serum in 1x PBS for 2 h. For the staining of neurons expressing EYFP, we used the chicken anti-GFP antibody (1:500, Abcam) overnight at 4°C. After the slices were washed three times in 1x PBS, goat anti-chicken 488 (1:500, Thermo Fisher Scientific) and DAPI (1:10,000 of 5 mg/mL, Cell Signaling Technology) were applied for 2 h at room temperature. Images were taken using a Leica SP8 X confocal system.

### Single-molecular fluorescent *in situ* hybridization

We performed single-molecular fluorescent *in situ* hybridization (smFISH) on fresh frozen sections using RNAScope Multiplex Reagent Kits (ACDBio, United States). We cut the frozen sections of 20 μm thickness in the cryostat within 30 min. The frozen sections were fixed with 4% PFA for 15 min, and then dehydrated in an ethanol series. H_2_O_2_ were employed to process the slices for 10 min, and then washed it in 1xPBS. Protease III were added and incubated at room temperature for 20 min. In our study, the RNA probes Slc17a6, Slc32a1, and EGFP were used. Next, slices were incubated with the RNA probes in the Hybez humidification incubator for 2 h and 30 min at 40°C, then washed with ACD wash buffer, and next incubated in the reagent AMP1-FL and AMP2-FL for 30 min, then AMP3-FL for 15 min. DAPI were applied for 10 min at room temperature. We used a Leica SP8 X confocal system to obtain images.

### Statistical analysis

Data are presented as the mean ± SEM. Statistical *p* values corresponded to the following significance levels: ^*^p < 0.05, ^**^p < 0.01, ^***^p < 0.001, and ^****^p < 0.0001. The data were analyzed using unpaired Student’s *t*-test in Figures 2E,F,I,J,M,N, 3D,G and Supplementary Figure S3B. Two-way ANOVA with Bonferroni’s multiple comparisons test was used in Figures 4D,E,I,J, 5D,E,I,J and Supplementary Figures S3D,E,G,H. All graphs and data analyses were performed using the GraphPad Prism 8 program.

## Data availability statement

The original contributions presented in the study are included in the article/Supplementary material, further inquiries can be directed to the corresponding author.

## Ethics statement

The animal study was reviewed and approved by Institutional Animal Care and Use Committee at Shanghai Jiao Tong University School of Medicine (Protocol A-2019-060).

## Author contributions

JH and AL designed the project and wrote the manuscript. AL performed the experiments and data analyses. YC helped for the smFISH and anxiety-related behavioral tests. All authors contributed to the article and approved the submitted version.

## Funding

This work was supported by grants from the Ministry of Science and Technology of the People’s Republic of China (2021ZD0202800/01) and the Natural Science Foundation of China (32070957).

## Conflict of interest

The authors declare that the research was conducted in the absence of any commercial or financial relationships that could be construed as a potential conflict of interest.

## Publisher’s note

All claims expressed in this article are solely those of the authors and do not necessarily represent those of their affiliated organizations, or those of the publisher, the editors and the reviewers. Any product that may be evaluated in this article, or claim that may be made by its manufacturer, is not guaranteed or endorsed by the publisher.
