# Supplementary figures and images for: Neurons innervating both the central amygdala and the ventral tegmental area encode different emotional valences
Source: Front Neurosci. 2023 May 5;17:1178693. doi: 10.3389/fnins.2023.1178693 (PMC10196062; doi:10.3389/fnins.2023.1178693)

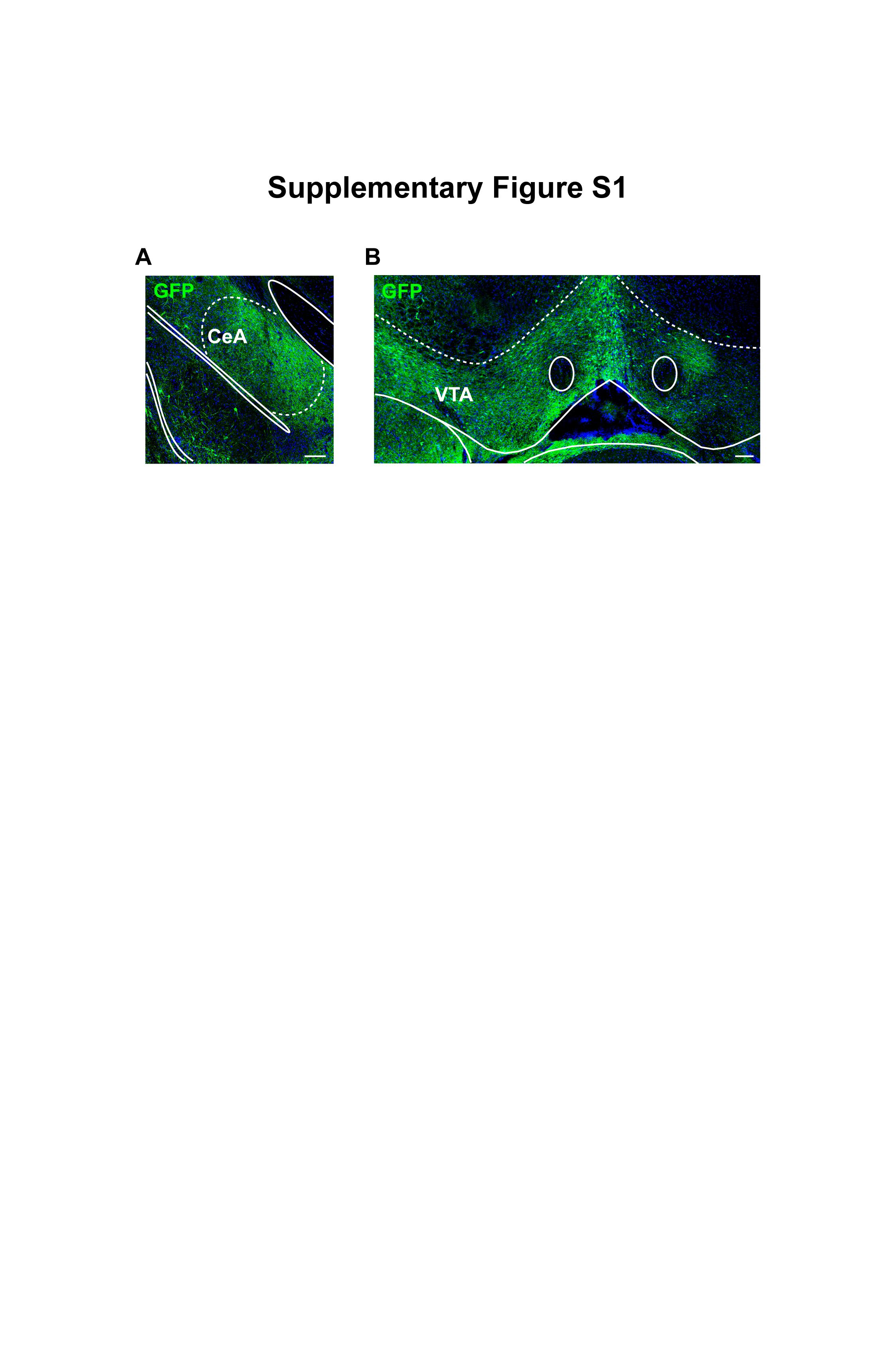

Supplement: Supplementary file 1 [file Image_1.JPEG]

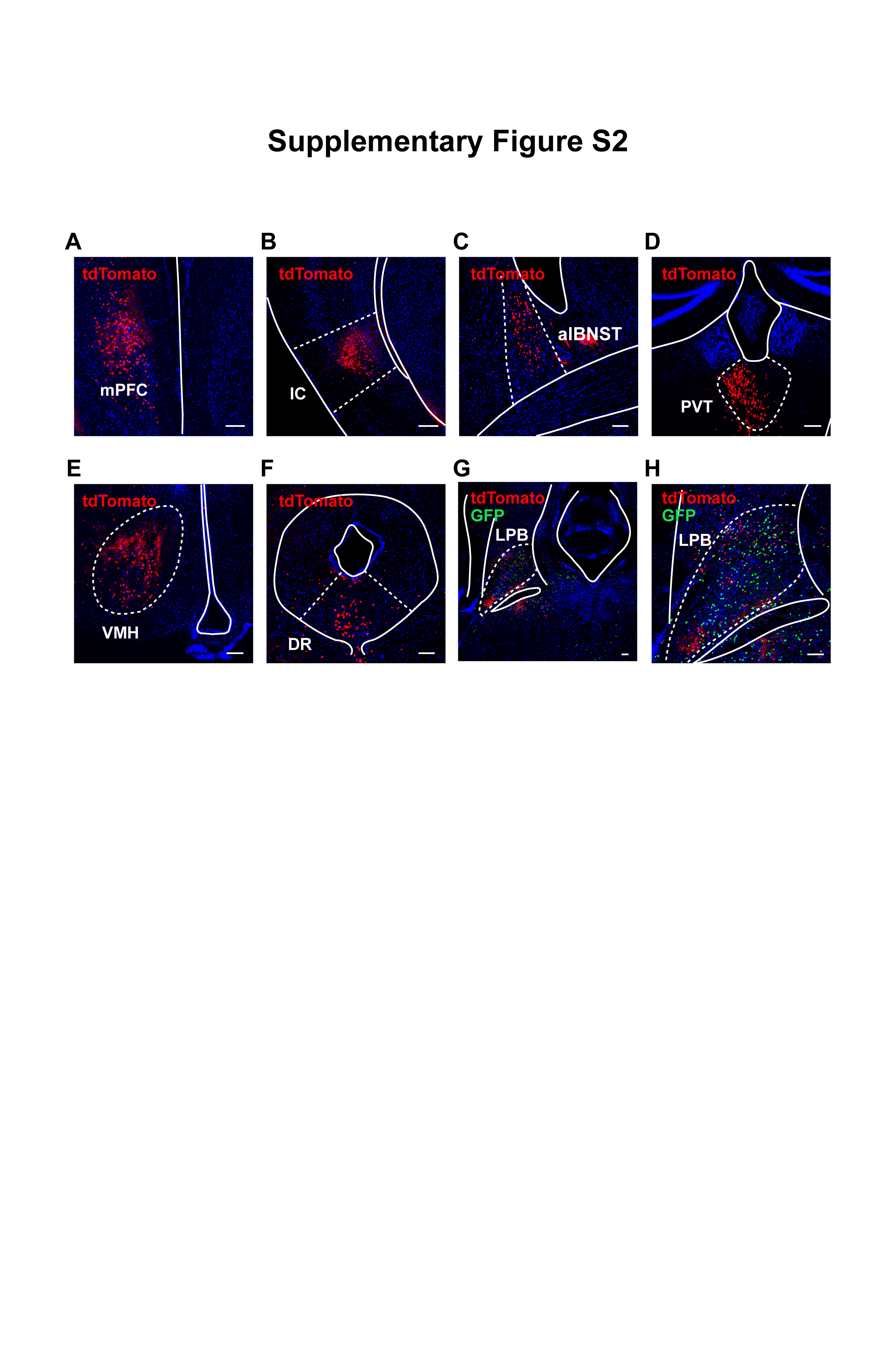

Supplement: Supplementary file 2 [file Image_2.JPEG]

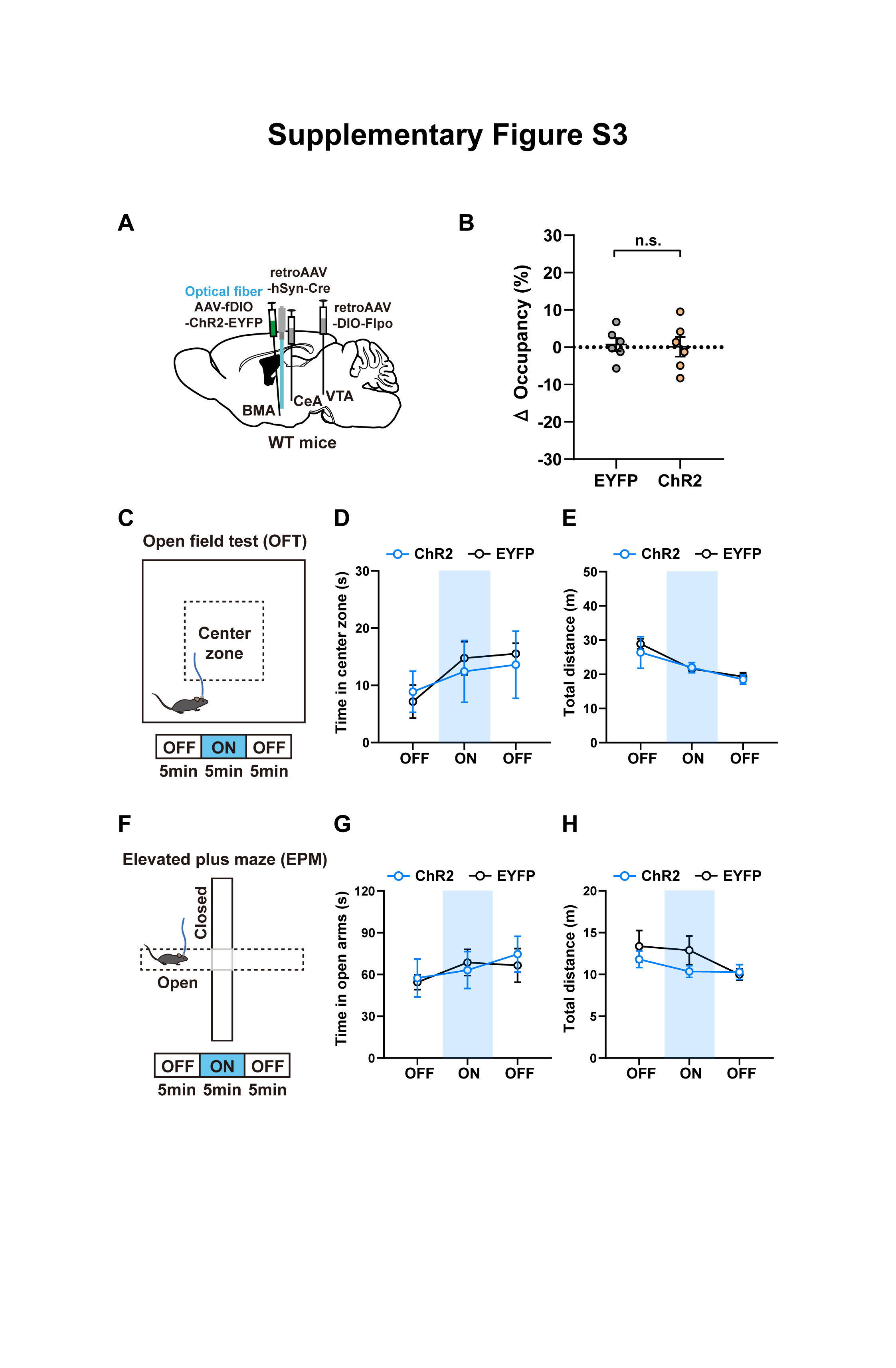

Supplement: Supplementary file 3 [file Image_3.JPEG]

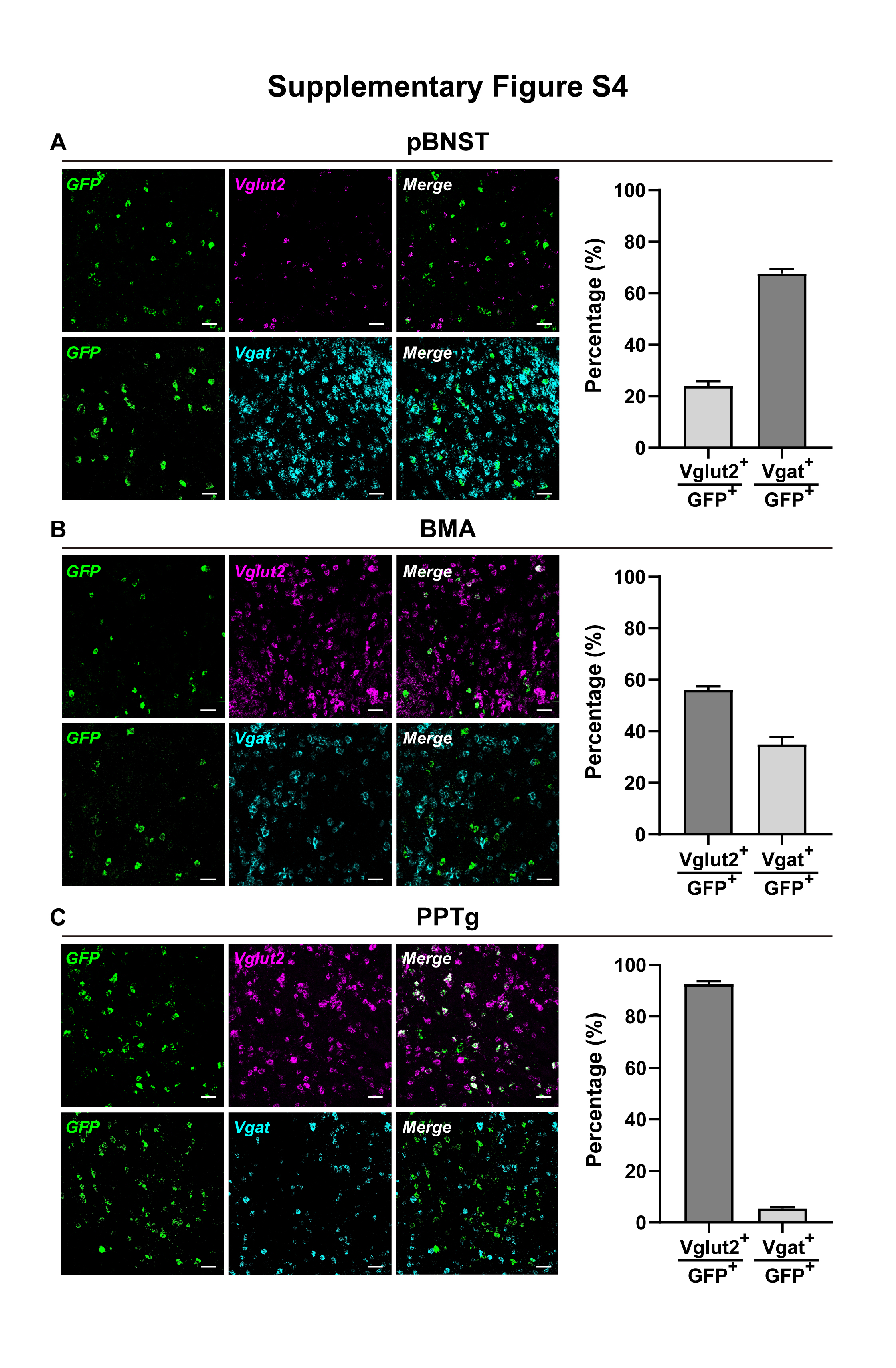

Supplement: Supplementary file 4 [file Image_4.JPEG]
