# Supplementary material for: Neurons innervating both the central amygdala and the ventral tegmental area encode different emotional valences
Source: Front Neurosci. 2023 May 5;17:1178693. doi: 10.3389/fnins.2023.1178693 (PMC10196062; doi:10.3389/fnins.2023.1178693)
Supplement: Supplementary file 5 [file Presentation_1.pdf]

Supplementary Figure S1. The projection of GFP-positive neurons to the CeA and the VTA

The GFP-labeled axons in the CeA **(A)** and VTA **(B)**. Scale bars: 100 μm.

Supplementary Figure S2. The methodological validity for using the IS reporter mice

(A-F) CeA-projecting tdTomato-positive neurons in the mPFC (A), the IC (B), the alBNST (C), the PVT (D), the VMH (E), and the DR (F). Scale bars: 100 μm. **(G, H)** The GFP-positive neurons and tdTomato-positive neurons in the LPB. Scale bars: 100 μm.

Supplementary Figure S3. Activation of BMA neurons innervating the CeA and the VTA has no effect on conditioned place testing and anxiety-like behavior

(A) A schematic diagram of the viral injection and fiber implantation sites. **(B)** Percent changes in the occupancy time on the stimulation side after conditioning (after conditioning - before conditioning) (n = 6 mice for each group). **(C)** Experimental procedure for intermittent optogenetic activation of ChR2-expressing neurons in the open field test. **(D, E)** Optogenetic activation of ChR2-expressing neurons in the BMA did not affect the time mice spent in the center zone and locomotor activity of mice in the open field test (n = 5 mice for each group). **(F)** Experimental procedure for intermittent optogenetic activation of ChR2-expressing neurons in the elevated plus maze test. **(G, H)** Optogenetic activation of ChR2-expressing neurons in the BMA did not affect the time mice spent in the open arms and locomotor activity of mice in the elevated plus maze test (n = 5 mice for each group). Data are presented as mean ± SEM.

Supplementary Figure S4. The types of neurons that innervate the CeA and the VTA (excitatory vs. inhibitory)

(A) Expression of *GFP*, *Vglut2*, and *Vgat* mRNAs with smFISH in the pBNST. Scale bars: 50 μm. 24.1% ± 1.8% and 67.7% ± 1.7% of GFP-positive neurons in the pBNST expressed *Vglut2* and *Vgat*, respectively (n = 3 mice). **(B)** Expression of *GFP*, *Vglut2*, and *Vgat* mRNAs with smFISH in the BMA. Scale bars: 50 μm. 56.1% ± 1.5% and 34.9% ± 3.0% of GFP-positive neurons in the BMA expressed *Vglut2* and *Vgat*, respectively (n = 3 mice). **(C)** Expression of *GFP*, *Vglut2*, and *Vgat* mRNAs with smFISH in the PPTg. Scale bars: 50 μm. 92.5% ± 1.2% and 5.5% ± 0.5% of GFP-positive neurons in the PPTg expressed *Vglut2* and *Vgat*, respectively (n = 3 mice). Data are presented as mean ± SEM.
